# Electroencephalogram-based pharmacodynamic measures: a review 

**DOI:** 10.5414/CP201484

**Published:** 2012-02-03

**Authors:** Michael Bewernitz, Hartmut Derendorf

**Affiliations:** Department of Pharmaceutics University of Florida, Gainesville, FL, USA

**Keywords:** electroencephalogram, EEG, drug, pharmacokinetic, pharmacodynamik, evoked potential, bispectral index

## Abstract

Pharmacokinetics and pharmacodynamics can provide a useful modeling framework for predicting drug activity and can serve as a basis for dose optimization. Determining a suitable biomarker or surrogate measure of drug effect for pharmacodynamic models can be challenging. The electroencephalograph is a widely-available and non-invasive tool for recording brain-wave activity simultaneously from multiple brain regions. Certain drug classes (such as drugs that act on the central nervous system) also generate a reproducible electroencephalogram (EEG) effect. Characterization of such a drug-induced EEG effect can produce pharmacokinetic/pharmacodynamic models useful for titrating drug levels and expediting development of chemically-similar drug analogs. This paper reviews the relevant concepts involved in pharmacokinetic/pharmacodynamic modeling using EEG-based pharmacodynamic measures. In addition, examples of such models for various drugs are organized by drug activity as well as chemical structure and presented.

## Introduction

Pharmacokinetics and pharmacodynamics are fields of study which aim to provide a means to predict drug activity. Pharmacodynamic effect models are designed to bypass the need to collect and assay fluid samples (e.g., plasma, urine, microdialysate) by predicting underlying drug concentration based on the measured effect. When linked with an appropriate pharmacokinetic model, the resulting pharmacokinetic/pharmacodynamic model can provide a meaningful representation of the drug effect profile over time. Pharmacokinetic/pharmacodynamic models can provide a safe, rapid, and inexpensive method to help ensure that a drug’s concentration remains within the safe, therapeutic range. 

The electroencephalograph has demonstrated use as a non-invasive, readily accessible tool for obtaining a surrogate measure for pharmacodynamic modeling in the emergency room as well in clinical research. General anesthetics, benzodiazepines and opioids are among the most common classes of drugs which generate an electroencephalogram (EEG) effect that can be related to drug concentration. Given the vast amount of post-synaptic potentials which contribute to each channel of an EEG recording, the large amount of multi-channel data obtained at hundreds if not thousands of samples per second, as well as inherent limitations of the modality, extracting drug-related effects can require sophisticated signal processing methods. Utilization of EEG as a surrogate effect can provide a non-invasive, rapidly-responding measure that can be readily applied at most clinical research and medical treatment facilities. 

This review will begin with an outline of pharmacokinetic/pharmacodynamic modeling in Section 1. In Section 2, an introduction to EEG is given with respect to clinical and research applications. Section 3 will overview EEG processing methods. Examples of EEG-based pharmacodynamic models for various drug classes will be presented in Section 4. Section 5 contains final remarks and the future direction of EEG-based pharmacodynamic modeling. 

## 1. Pharmacokinetic/ pharmacodynamic modeling

In order to put the EEG pharmacodynamic concepts into proper context, a brief review of pharmacokinetics, pharmacodynamics, and coupled pharmacokinetic/pharmacodynamic models will be presented. A drug is a chemical agent which can be used for or to assist with alleviating, curing, diagnosing or preventing a disease [1]. If a drug is taken orally, the set of processes involved in breaking down the drug dosage compound in preparation for absorption is referred to as the pharmaceutical phase. Upon disintegration of an oral dosage form or administration of a non-oral route (e.g. intravascular or intravenous, intramuscular, intraperitoneal, sub-cutaneous, topical, etc.) the pharmacokinetic phase of drug action begins. 

### 1.1 Pharmacokinetics

The pharmacokinetic phase is comprised of the transportation and chemical changes of the drug. In lay terms, pharmacokinetics is the study of “what the body does to the drug”. Pharmacokinetic models describe the time course of observed drug concentrations within the body, often in the plasma or tissue fluid. The major processes considered in pharmacokinetic modeling are absorption, distribution and elimination of the drug. Absorption is the transport of the drug into the blood stream. Distribution is the transport of the drug from the blood into the tissues. Elimination includes biotransformation, metabolism and excretion processes that diminish drug concentration levels in the body. 

Pharmacokinetic models are formulated to a particular drug treatment scenario (where each scenario may vary drastically depending on a number of treatment parameters). For example, a drug’s concentration profile can vary depending on the route of administration (e.g., i.v. bolus, i.v. infusion, oral etc.), the presence of certain diseases or disorders (meningitis can affect blood-brain barrier drug transport of opioids), and can even vary with genetic expression [2]. In addition, different drugs display kinetic behavior that can vary greatly in the degree of complexity. Mathematically, the simplest modeling scheme is the one-compartmental approach, which assumes the absorbed drug rapidly distributes to all tissues in the body and elimination follows exponential decay. As the drug does not usually appear to evenly distribute into only one compartment after absorption, a more common approach is to utilize a two or three compartment model [1]. For example, in addition to the elimination rate term, a two-compartment framework includes a hypothetical peripheral compartment to model the distribution. Additional parameters can be included to model more complex drug kinetic behavior. 

Modern pharmacokinetic modeling takes into account intra-individual and inter-individual variation among a study population. For example, the non-linear mixed effects modeling technique can form models for predicting drug concentration in a patient population. Such models may incorporate fixed effects (e.g., creatinine clearance, patient age, body mass and sex) as well as random effects to account for various confounding factors. 

### 1.2 Pharmacodynamics

Pharmacodynamic models describe the relationship between drug concentration and drug activity. In lay terms, pharmacodynamic models describe “what the drug does to the body”. The effect of a drug can be desirable by resulting in treatment efficacy, undesirable resulting in side effects (e.g., nausea, dizziness) or toxic and resulting in impaired organ function and/or death. Drugs undergo various binding processes to interact with biological target sites including hormone and neurotransmitter receptors, enzymes, carrier molecules, ion channels, idiosyncratic targets (such as metal ions, surfactant proteins, gastrointestinal contents) and nucleic acids [3]. 

The binding process may cause a direct effect in the receptor cell(s) or transduce a signal to produce a downstream pharmacological effect. Reversible drug effects can be related to plasma drug concentration, often using the sigmoid E_max_ equation (Equation 1) [4]. 

Equation 1: the sigmoid E_max_ equation, a variation of the Hill equation [5]. E is the measured pharmacologic effect, E_0_ is the baseline effect prior to drug administration, E_max_ is the maximum effect the drug can elicit, EC_50_ is the concentration which produces 50% of the maximum drug effect, C is the drug concentration at the receptor site, and γ is an exponent regulating the shape of the concentration-effect curve. 

The receptor concentration in the sigmoid E_max_ equation is often very difficult if not impossible to determine in clinical pharmacodynamic studies. As an empirical alternative, the unbound plasma concentration is often measurable for a wide range of drug concentrations. If the drug concentration at the receptor site is proportional to the plasma concentration then the plasma concentration may be utilized in the sigmoid E_max_ . 

### 1.3 Application of pharmacokinetic/pharmacodynamic models

Pharmacokinetic-pharmacodynamic models can be useful for dose optimization. In other words, such models can help establish dose regimens designed to produce plasma concentrations above the minimum effective concentration and below the minimum toxic concentration for the maximal time that can be practically achieved. Such a model can be especially useful in expediting drug development. 

Each pharmacodynamic model must employ a biomarker which is a suitable surrogate measure of the drug effect. For example, blood pressures may be used in pharmacodynamic models of hypertension treatment. In addition, the time above minimum inhibitory concentration (MIC) may serve as a pharmacodynamic measure for antibiotics. 

Some drugs produce an effect on neuronal firing patterns that may manifest as bulk alterations in brain wave field potential recordings. Such recordings are called electroencephalograms, or EEGs. In order to better understand the advantages and challenges of EEG-based pharmacodynamic measures, it is necessary to review the fundamentals of EEG. 

## 2. Electroencephalogram (EEG) in medicine and research

The spontaneous continuous electrical activity of the brain was first discovered in 1875 by a physician named Richard Caton [6]. Hans Berger is considered to be the founder of electroencephalography due to his 1929 report describing spontaneous electrical activity in humans including characterization of the α-band and β-activity [6]. In addition, in a 1932 publication Berger described four photographic EEG segments recorded from a patient recovering from postictal confusion [6]. 

EEG is one of the most common tools for diagnosis and evaluation of neurological disorders. For example, EEG recordings are the foundation for epileptic diagnosis as well as classification of epileptic seizures [7]. Electrical field potentials can be detected using electrodes located in the proximity of nervous system tissue. Field potentials originate from neuronal and glial cell membrane potential changes. When an excitatory afferent fiber is stimulated, the resulting influx of cations (e.g., sodium) can lead to post-synaptic membrane depolarization. This process is referred to as an excitatory post synaptic potential (EPSP). Propagation of membrane depolarization produces both intracellular currents as well as extracellular currents. 

A negative field potential charge appears in a nearby electrode due to extracellular currents (cationic influx) whereas a positive charge (cationic outflux) appears in a more distant electrode [7]. Similarly, during an inhibitory post synaptic potential (IPSP), inhibitory afferent fiber stimulation induces cationic outflux that is perceived as a positive charge by a nearby electrode. These electrical events from large populations of brain cells are detected as a summed potential at a nearby electrode. 

EEG is most often acquired from scalp-EEG electrodes. While surgically-implanted electrodes can record from the surface of the cortex or from subcortical regions (such as the hippocampus) for research or clinical purposes, such invasive electrode locations cannot practically be applied in large-scale pharmacokinetic/pharmacodynamic studies. For this reason, this article will focus on EEG recordings acquired from scalp electrodes. 

The international 10 – 20 system has been developed in order to standardize electrode placement. This system designates electrode positions using the nasion and inion as reference points. The electrode locations are then spaced proportionally (e.g., 10 – 20% intervals) with respect to the distance between the nasion and inion [8]. 

EEG acquired from the scalp electrodes is susceptible to artifact from a range of sources. Skull muscle exertion, eye blinks and alternating current noise are some of the most common sources of contaminating signals. These particular artifacts can obscure useful neural firing patterns. 

Proper placement of a reference electrode (e.g., on an earlobe) can help reduce certain EEG artifacts. In order to understand this method, one needs to understand the nature of the EEG signal. It is important to note that the voltage recorded by the electroencephalograph is actually the voltage difference between a recording electrode and a reference electrode. In this manner, artifacts that are present at both the electrode of interest and the reference electrode are significantly reduced when the voltage difference between the two sites is recorded. This is called common mode rejection. 

Upon EEG acquisition, additional filtering is often required to enhance signal quality. Numerous methods are available to significantly reduce the presence of artifact, some of which are built into the EEG acquisition hardware. In order to understand the nature of such filtering processes (described in Section 3.3), a brief overview of EEG processing techniques is necessary. 

## 3. EEG processing techniques

This section will first review some common EEG analysis methods and then describe their practical application (such as pre-processing and feature extraction). A feature can be defined as a summary measure that characterizes salient aspects of a signal such as δ-band EEG spectral power or evoked potential amplitude [9]. This section is subdivided into two sub-sections; the first outlines mathematically-based EEG features, the second refers to biologically meaningful EEG features. For a more in-depth review of quantitative EEG analysis methods, please see Thakor and Tong [10]. For a more in-depth review of issues regarding clinical applications of EEG-based pharmacokinetic-pharmacodynamic modeling, please see Barbanoj et al. [11]. 

### 3.1 Mathematically-based EEG features

This section examines the application of various signal modeling techniques. 

****3.1.1 Discrete Fourier transform****

The Fourier transform has been extensively applied in biology, mathematics, and physics. The development of the streamlined Fast Fourier Transform (FFT) has vastly reduced computational complexity over the original formulation [12]. More recently, further developments have been implemented into a subroutine library called FFTW [13]. This software package provides a user with various heuristic approaches to optimize the FFT algorithm for each unique computer platform and dataset combination. Advancements such as these and others provide the opportunity for rapid offline analysis and even real-time analysis of signals such as EEG. 

The Fourier transform decomposes a signal into components that can be combined linearly. The analysis is founded on the concept that a time series can be properly represented by the linear summation of sine waves of different amplitudes, frequencies, and phases. For a discrete signal, x_n_ = {x_0_,..., x_N–1_}, where n = 0, ..., N – 1 represents the sampling time indices with a total of *N *samples, the discrete Fourier transform (DFT) is represented as: [Fig Equation2]

Equation 2, discrete Fourier transform 

with the inverse discrete Fourier transform expressed as:[Fig Equation3]

Equation 3: inverse discrete Fourier transform, where X_k_ is the spectrum of *x* at frequency *k*, *i* is the square root of negative 1, and π is the ratio of the circumference to the diameter of a circle. Though the Fourier analysis can accurately model periodic waveforms in stationary signals, this technique is not able to extract transient features. In other words, Fourier analysis is intended for signals with a steady rhythm. Since EEG signals often display rapid changes in rhythm, a common strategy is to split the recording into contiguous segments and apply Fourier analysis to each segment. In this manner, the so-called “windowed” Fourier analysis provides an approximation of frequency content in each epoch. The “windowed” discrete Fourier analysis is a step used in many EEG analysis techniques, such as spectral edge frequency and cordance. 

****3.1.2 Spectral edge frequency****

The spectral edge frequency measure attempts to summarize a signal’s spectrum with a single number. The spectral edge frequency is the frequency that is greater than a specified fraction f (e.g., f = 0.95) of the total signal power. The “edge frequency” is determined by first performing a Fourier analysis and forming a histogram of the power versus frequency. If f = 0.95, for example, then the spectral edge frequency is the frequency that resides above 95% of the total signal power [14]. 

****3.1.3 Cordance****

Cordance is a derived measure which produces an index based on normalized and non-normalized EEG spectral features [15, 16, 17]. A detailed explanation of the cordance calculation procedure is outlined in Leuchter et al. [18]. Briefly, after rejecting channels heavily afflicted with artifact, the first step is to estimate EEG power in windows less than 30 seconds in duration at individual electrode sites from a bipolar montage dataset using the Fourier transform. The absolute power at electrode “X” is estimated by calculating the mean absolute power for all electrode pairs involving electrode “X”. Relative intra-channel EEG power in overlapping 4-Hz bands is then calculated by dividing the band power by the total signal power. The absolute as well as the relative power levels for each frequency band are then spatially normalized across all electrode sites using z-scores. The cordance of a particular EEG band at a particular recording site is designated as the sum of the normalized absolute and normalized relative powers for that band and site. 

****3.1.4 Wavelet analysis****

Wavelet analysis is a signal processing method introduced by Grossman and Morlet [19] that models a time series signal in terms of multiple components, each a translated and scaled version of a “mother” wavelet function. In some aspects wavelet analysis is similar to Fourier analysis, which uses sinusoids to model a signal. Instead of sinusoids, wavelet analysis models the signal with a user-defined waveform function called a wavelet with a mean value of 0 and an effectively limited duration [20]. While Fourier analysis is only intended for constant rhythms, wavelet analysis can also characterize time-varying rhythms. Specifically, wavelet analysis can characterize breakdown points, complex trends, discontinuities in higher derivatives, as well as self-similarity [20]. For the selected mother wavelet function Ψ*(t)*, the signal at time scale *a*, position *b*, and time *t* is represented by:[Fig Equation4]

Equation 4, where *a *∈* R* ^+^ and *b* ∈ *R* [21]. If *x(t)* is a discrete time series signal, then a continuous wavelet transformation at scale *a*, position, *b*, and time *t* is defined as:[Fig Equation5]

Equation 5, where 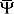 represents the mother wavelet’s complex conjugate and *a *∈* R* ^+^ and *b* ∈ *R* [21]. When the scaling factor *a* takes on low values the wavelet is compressed (becomes more “spikey”) and may provide a closer approximation of rapid amplitude changes and high frequency rhythms. When the scaling factor *a* takes on higher values the wavelet is stretched (becomes less “spikey”) and may provide a closer approximation of the gradual amplitude changes and low frequency rhythms. The position parameter *b* translates the wavelet in time. Wavelet analysis has demonstrated satisfactory modeling of complex non-stationary signals such as EEG [22, 23, 24, 25]. 

There are additional time-frequency transforms that are commonly utilized in EEG processing. An exhaustive list of these transformations is beyond the scope of this paper. Please see Rampil [26] for more details on time-frequency transformation-based techniques commonly applied in EEG signal analysis. 

****3.1.5 Entropy****

Entropy is a fundamental concept of information theory. Details of information theory are beyond the scope of this paper, however, a theoretical basis relevant to EEG signal processing can be found in Pereda et al. [27]. A common and practical interpretation of signal entropy is (roughly speaking) the quantification of signal regularity [28]. 

A relationship between plasma drug concentration and entropy is evident for numerous drugs using various entropy estimation methods. For example, some methods that have demonstrated a pharmacodynamic link with effect-site concentrations include permutation entropy [29, 30], Shannon entropy [31] and spectral entropy [32, 33, 34, 35]. This review will focus on a popular method called approximate entropy (ApEnt). 

**3.1.5.1 Approximate entropy**

ApEnt is a statistic developed to quantify system complexity and regularity from an observed time series dataset [36]. The ApEnt measure has demonstrated the ability to quantify system complexity using as few as 1,000 data points based on theoretical analyses of stochastic and deterministic chaotic processes [37] as well as clinical applications [36, 38]. 

Approximate entropy is estimated as follows. Let *U* represent a signal with *N* samples. For a positive integer *m* and a positive real number *r*f, create a number of vectors equal to *N – m +* 1, where each vector is defined as *x*m*(i) = {u(i),u(i+1), ...,u(i+m–1)}*. For each *i*, where 1 ≤ 1 ≤ *N – m* +1, the quantity C^m^_i_ is calculated in the following manner:[Fig Equation6]

Equation 6, where *d* is the maximum absolute difference between each of the *m* respective scalar components of the vectors *x*m*(i), x*m*(j)* expressed as:[Fig Equation7]

Equation 7.

From here, the quantity Φ^m^ can be computed as:[Fig Equation8]

Equation 8:

and ApEnt is estimated as: [Fig Equation9]

Equation 9.

The variable *m* is the embedding dimension. The positive real number *r*f is a threshold distance between neighboring points which is usually assigned as a fraction of the signal’s standard deviation. In this context, the positive real number *r*f can be viewed as a filter for the process. 

The ApEnt of EEG has been used in pharmacokinetic/pharmacodynamic studies as a surrogate measure of anesthetic depth [29, 31, 38, 39, 40]. The ApEnt EEG measure has also been utilized in the pharmacokinetic and pharmacodynamic modeling of the opioid remifentanil [41]. 

****3.1.6 Bispectral index****

Since its release in 1996, the bispectral index monitor (BIS, Aspect Medical Systems Inc., Natick MA) has provided physicians with a ready-to-use EEG-based monitor for titrating and maintaining depth of anesthesia [42]. The purpose of this device is to help reduce anesthetic use, improve patient recovery time, and prevent patient recall. A recent study established that use of the BIS-guided anesthesia administration reduced the requirement for propofol by 1.30 mg/kg/h and volatile anesthetics by 0.17 minimal alveolar concentration equivalents [43]. Numerous review papers are available to discuss the BIS system at length [26, 42, 43, 44, 45]. The scope of this paper will focus on the aspects that are relevant to the field of pharmacodynamics. 

The Bispectral Index^®^ (BIS) is a numerical index derived from a proprietary processing method involving multiple EEG measures for use as a surrogate marker of anesthetic depth. The EEG measures are mathematically combined to obtain a numerical index which ranges from 0 to 100 [44]. This numerical index is derived from empirical correlations established between EEG features and patient vigilance states. 

The EEG measures applied to the BIS include burst suppression ratio, α/β-band power ratio (where α is the 8 – 13 Hz band, β is the 13 – 30 Hz band), and bicoherence (a measure of the degree of phase coupling among two frequencies within a single EEG channel) [46]. The burst suppression ratio is a time-domain feature describing the relative degree of electrical silence compared to slow waves. 

The bispectral index is typically obtained from 3 scalp EEG electrodes (for the BIS system) or 4 scalp EEG electrodes (for the BIS-XP system) that attach to the forehead with a self-adhering strip [44]. 

One of the most attractive features of the BIS system is its broad applicability. The BIS was developed based on a large volume of EEG data acquired during sedation and hypnosis from numerous patients under the effects of a wide range of anesthetic and sedative drugs [26]. However, concerns have been raised regarding the reliability of the BIS measure, particularly when multiple drugs are used simultaneously in anesthesia. For example, the BIS measure has failed to demonstrate sensitivity to hemodynamic fluctuations associated with consciousness change induced by intravenous pancuronium, fentanyl, and midazolam during endotrachial intubation and stenotomy [47]. In addition, multiple studies have reported different software versions (or omitted software version), and/or different statistical methods, making comparison of BIS studies difficult [44]. In addition, concerns have been expressed regarding a lack of explicit biological relevance for the BIS [46]. Despite these concerns, a range of pharmacodynamic studies have demonstrated a link between the EEG BIS and drug concentration. These studies are described in Section 4. 

****3.1.7 Current source density****

Current source density estimation is an analysis technique for localizing the source of electrical activity on EEG recordings. The low-resolution brain electromagnetic tomography (LORETA) current source density technique first presented by Pascual-Marqui et al. [48] estimates the electrical current spatial distribution throughout the brain volume. In this premier application, occipital lobe current source distributions were estimated from visual evoked-potential EEG recordings. The original method has since been improved to produce standardized current source density images with zero localization error [49]. While the spatial resolution is considered low, this method is able to take advantage of the high time resolution of EEG recordings. This method has since been applied to characterize the effects of various psychoactive drugs on the spatial distribution of electrical current sources in the brain [50, 51, 52, 53, 54, 55, 56]. Saletu et al. [57] describe a “key-lock” principle based on two important observations involving current source density maps. First, various mental disorders produce EEG maps (as measured using LORETA) that differ statistically from each other as well as from normal controls. Second, psychopharmacological drugs have been shown to induce typical and significant changes in brain function compared to placebo wherein many variables are in opposition to the observed EEG differences between psychiatric and normal patients. Thus, it may be possible to treat a mental disorder using this key-lock principle to select the optimal drug to normalize the deviant brain function. 

### 3.2 Useful EEG patterns in clinical neurology

Drugs which act on the central nervous system can produce complex effects that may be difficult to capture using mathematically-derived measures. Thus, in some situations a drug effect can be characterized more effectively by examining physiologically-based EEG waveforms. Such waveforms can be generated spontaneously and others may be a provoked neural response. Basic features of these waveforms, such as total duration, rate of occurrence, or peak amplitude may be used as measures of effect. Such measures can provide insight into the drug’s effect on the underlying neuronal processes. 

****3.2.1 Interictal spike and wave discharges****

The interictal (between-seizure) spike and wave discharge is a seizure-related EEG pattern that can be useful in pharmacodynamic EEG studies. The EEG interictal spike discharge stems from synchronized pyramidal neuronal action potentials from a hyperexcitable region of the neocortex which can be the origin for focal seizures [58]. In addition, an extended neuronal hyperpolarization period following the action potentials is the cellular mechanism that produces an EEG slow wave subsequent to the spiking [58]. Interictal spikes have been used in EEG pharmacodynamic studies of midazolam and diazepam [59] as well as carbamazepine and stiripentol [60]. Caution must be exerted when using this EEG waveform as a pharmacodynamic measure since seizures can initiate after interictal spike and wave discharges or in their absence in both animal models and humans [58]. 

****3.2.2 Ictal spike and wave discharges****

Seizure models are a traditional method for early evaluation of potential epilepsy treatment drugs. Such models include provocation of seizures in animals using maximal electroshock [61] or pentylenetetrazol [62]. Drugs that prevent the seizing response induced by these two proconvulsant methods are presumed to have an antiepileptic effect. Additionally, various convulsants such as the GABA antagonist bicuculline, the chloride-channel blocker picrotoxin, and the glycine antagonist strychnine may also be used to identify the antiepileptic mechanism [63]. 

EEG is useful for the evaluation of anticonvulsant drug action in terms of electroencephalographic seizures. The seizures observed in such studies may be spurred by chemoconvulsants (in animal studies) or may occur naturally as a result of the epileptic condition (in human patients). Antiepileptic drug effects on ictal EEG spike and wave discharges have been applied in antiepileptic pharmacodynamic studies [64, 65, 66, 67]. 

An additional EEG pharmacodynamic measure applied in antiepileptic medication assessment is the frequency of seizure occurrence [63]. The raw seizure count may be a crude measure of antiepileptic drug effect, but it is used more commonly than other EEG measures [63]. 

****3.2.3 Evoked potentials****

Evoked potentials (EPs) are biologically meaningful EEG patterns produced in response to specific stimuli. The EP patterns observed subsequent to the stimulation represent the neural activities associated with processing of the perceived stimuli. Due to the intra-individual and inter-individual consistency, EPs can provide useful information about specific brain functions which can be related to disorders and/or drug activity [68, 69]. Evoked potentials are obtained by recording EEG while exposing the patient to multiple repetitions of a novel stimulus. The multiple trials are then processed (e.g., time averaged) to help eliminate background noise and accentuate the neural response signal. 

EPs can be broadly categorized according to their onset latency. The earlier (exogenous) components occur within about 75 ms and are elicited by the stimulus [70]. The longer latency EP components differ in many aspects. The long-latency (endogenous) components are generated above the brainstem and generally have higher amplitudes and lower frequencies than exogenous EPs [71]. Endogenous EP components can start at about 75 ms [70]. In contrast to exogenous EPs, endogenous EPs are not markedly affected by stimulus parameters such as frequency and intensity [71]. Also, the endogenous EP components are sensitive to the mental status of the patient. For example, exogenous EPs can be detected while the subject is under anesthesia, whereas endogenous EPs such as the P300 require the patient to be awake and alert [71]. The latency of endogenous EPs reflects the neural activity time course whereas the amplitude reflects the extent of neural resource allocation to the specific cognitive processes [69]. 

The P300 evoked potential is a large, broad, positive component of the EP that typically peaks at 300 ms or more after the onset of a rare, task-related stimulus [69]. The P300 can be readily observed when applying an “oddball” paradigm wherein subjects detect and respond to an unexpected target stimuli presented at random times amongst non-target stimuli [70]. Stimuli of various modalities can be used to elicit a P300 evoked potential [71]. One important requirement for producing a P300 evoked potential is that the subject must be paying attention to a series of stimuli when the unexpected event occurs [70]. The P300 amplitude can be affected by attentional and perceptual variables whereas the physical parameters of the stimuli have little or no affect [69]. Furthermore, as the stimulus processing complexity for a task increases, so does the P300 latency which can range from ~ 250 to 1,000 ms [69]. Anderer et al. [52] illustrate the P300 utility in a study where they demonstrated that the auditory evoked P300 latency was delayed and amplitude was reduced in patients with age-associated memory impairment as well as the group of older patients. 

Evoked potentials can be introduced through various sensory pathways. The sensory methods most commonly applied are auditory, visual and somatosensory evoked potentials. The following section will present a brief overview of the different evoked potential modalities. 

**3.2.3.1 Visual evoked potentials**

Visual evoked potentials measure the brain’s response to visual stimuli. This is achieved by recording EEG while having the patient watch a video pattern broadcast on a monitor or projector. One challenging aspect of this method is accounting for the effects of interindividual eyesight variability on visual EP’s among multiple patients. 

*3.2.3.2 Somatosensory evoked potentials*

**Figure 1:**
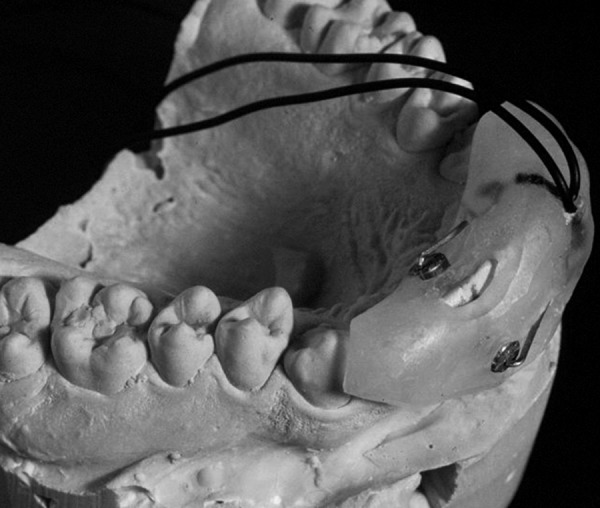
Schematic presentation of an electrode assembly used to elicit painful stimuli via electrical pulses.

Somatosensory evoked potentials (SSEP) involve a tactile stimulation, e.g., using electrical pulse stimulation of wrists or ankles. Suri et al. studied the pharmacokinetic-pharmacodynamic effects of codeine [72] as well as ibuprofen and flurbiprofen [73] using electrical pulses to stimulate tooth pulp and recording the SSEP as an objective measure of pain response. An example of a tooth pulp electrical stimulation apparatus can be seen in Figure 1. 

One downside to this method is that the discomfort associated with electrical stimulation may dissuade patients from SSEP studies. Likewise, other SSEP methods such as mechanical or laser stimulation produce unpleasant sensations but also have issues with repeatability (both methods may injure the patient’s skin and thus alter the response to the stimuli). 

*3.2.3.3 Auditory evoked potentials*

The auditory evoked response is one of the most commonly applied EP paradigms for drug effect studies. This experimental paradigm involves placing a small audio speaker into the subject’s ear and repeatedly playing a brief clicking sound. Auditory evoked potential response is categorized as originating from the brainstem (earliest response), midlatency and long latency [74]. An example application is the use of mid-latency auditory EP response to monitor depth of anesthesia [35, 75]. In addition, lamotrigine and topiramate are examples of drugs that elicit evoked potential as well as EEG effects [76]. 

Examination of such biologically-significant EEG waveforms can provide biological relevance to observed drug effects. However such EEG features may not be sensitive to every drug, may be difficult (or unethical) to induce in humans, and may require extensive manual EEG review (e.g., may require dozens of EEG-reviewer hours to score a single hour of EEG). In particular, desired EEG features may be difficult to detect in the presence of noise and artifact. While EEG artifacts can originate from many external sources, it is important to note that the patient’s own body can extensively contaminate recordings. One such biological artifact source is an eye blink (which will be revisited in Section 3.3.1). 

In order to enhance the quality of the desired EEG waveforms, the raw EEG signal is subjected to a cleansing routine prior to analysis. Standard EEG as well as evoked potentials can both benefit from such filtration and artifact rejection procedures. For these reasons, the first steps in quantitative analysis of standard EEG or evoked potentials is signal pre-processing. 

### 3.3 EEG pre-processing

In clinical as well as research settings, the raw EEG signal can be contaminated with artifact and noise. The EEG signal amplifier performs limited filtering functions as it amplifies the raw voltages. Such filtering may include high-pass filtering (frequencies lower than the “cut-off” frequency are significantly attenuated) and low-pass filtering (frequencies higher than the “cut-off” frequency are significantly attenuated). High-pass and low-pass filters are often set at or near 1 Hz and 70 Hz as this range contains the EEG activity of greatest interest [77]. 

Additional filtering techniques are usually applied prior to review or quantitative analysis. While the amplifier can filter the raw EEG signal, the amplifier filtration is usually limited in scope and typically performs a modest attenuation of obvious artifact. Software filtering is often performed on a case-by-case basis depending on what waveforms are of interest. 

Signal filtering routines are often applied in order to enhance the EEG signal-to-noise ratio. For example, electrical power supplied to run the laboratory equipment has an alternating current frequency that can (and usually does) leak into the EEG recording. In order to mitigate the presence of this electrical interference, a band-stop filter (also called a “notch” filter) is implemented to attenuate a narrow range of frequencies such as those imposed by the alternating current artifact. For example, most countries in the world supply electricity at 50 Hz (e.g., the European Union, most Asian countries) and thus EEG recordings in these countries would benefit from application of a 50 Hz notch filter. However, EEG recorded in other countries including the United States requires a notch filter centered at 60 Hz. 

**3.3.1 Eye blink artifact removal**

Eye blink artifacts can cause serious interference with the interpretation of scalp-EEG recordings. Eye blink artifacts can be a challenge to remove as traditional time and frequency domain filtering approaches can also distort waveforms originating from the brain. Many techniques have been implemented in order to mitigate eye blink artifact while minimizing the impact on standard EEG [78, 79, 80, 81, 82, 83, 84] as well as evoked potentials [85, 86]. 

Principal component analysis (PCA) is a useful data reduction technique which uses second-order statistics to transform multiple potentially correlated variables into fewer orthogonal (statistically unrelated) variables, called principal components. Under the assumption of a linear mixture of multiple signal components, PCA may be able to isolate unwanted EEG signal components (e.g., eye blink artifacts) and subsequently remove them from the EEG signal. One downside to PCA is the requirement that the principal components must be orthogonal. For real-life datasets such as EEG recordings, this assumption may not be accurate [84]. 

Independent component analysis (ICA) is a useful technique that has been utilized for the mitigation of eye blink artifact. ICA aims to statistically model a signal such as EEG as a linear combination of components which are as independent as possible [83]. 

The process of decomposing a signal that was “mixed” a priori in such an unknown manner is referred to as blind source separation (BSS). BSS refers to a collection of methods wherein a measured signal is treated as a mixture of multiple independent source signals. An example of this method is a study where Fitzgibbon et al. [84] were able to separate or “unmix” the EEG signal into multiple source signals using the PCA-based and ICA-based BSS algorithms, remove muscle and eye blink, and remix the remaining EEG components. While ICA treats different signals as statistically independent components, the second order blind interface (SOBI) method “decorrelates” the EEG signal components across multiple time lags [87]. Frank and Frishkoff [80] found that SOBI performed similar to two ICA algorithms at removing eye-blink artifact. 

**3.3.2 Muscle artifact removal**

Scalp-EEG recordings are also susceptible to electromyographic (EMG) artifacts originating from cranial muscle. EMG artifact can be troublesome to remove as various muscle groups can produce effects in numerous frequency ranges. For example, Goncharova et al. [88] observed that frontalis contraction produced artifact in the 20 – 30 Hz range whereas temporalis contraction produced artifact in the 40 – 80 Hz range. In addition, another study demonstrated EMG artifact occurring in the 0.25 – 32 Hz band with up to 70% occurring in 15 – 32 Hz in some subjects [89]. 

Muscle artifact has been addressed in numerous manners. Brunner et al. [89] have implemented a sleep-study algorithm that compares muscle activity in the 26.25 – 32 Hz band in a 4-second epoch against a rejection “threshold” of the activity found in the same frequency band of a localized 3-minute window. De Clercq et al. [90] outlined a method using canonical correlation analysis as a BSS technique to remove muscle artifact in EEG. When applied to ictal EEG, the canonical correlation analysis outperformed both a low-pass filter and an ICA-based technique [91]. Another study found that the algorithm for unknown source extraction (AMUSE) ICA approach was more effective than three other ICA algorithms at separating EMG from EEG during sleep [92]. McMenamin et al. [93] demonstrated that while within-subject epoch-based residualization EMG correction outperformed three other regression-based techniques using scalp-EEG, none of the methods performed well when applied in the spectral source space. 

## 4. Prominent drug classes in the realm of EEG pharmacodynamic modeling

This section provides a non-exhaustive overview of exemplar EEG-based pharmacodynamic modeling cases. 

### 4.1 Analgesics

**Figure 2.**
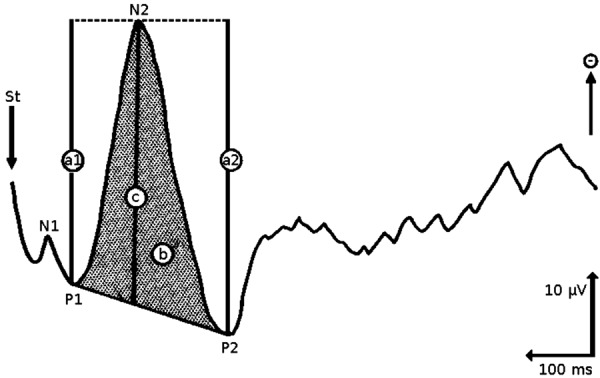
A procedure for the quantification of evoked potential data. “St” is the time of stimulus, “A1” and “A2” are peak to peak amplitudes with respect to P1 and P2 (respectively), “b” is the area under the N2 curve, and “c” is the measurement of the N2 peak with respect to P1 and P2 (figure adapted from [94]).

Analgesic drugs aim to reduce the perception of painful stimuli. One useful method for assessing analgesic effect is to use SSEPs as a pharmacodynamic measure. Figure 2 shows an example of an SSEP and some extracted features [94]. 

**Figure 3.**
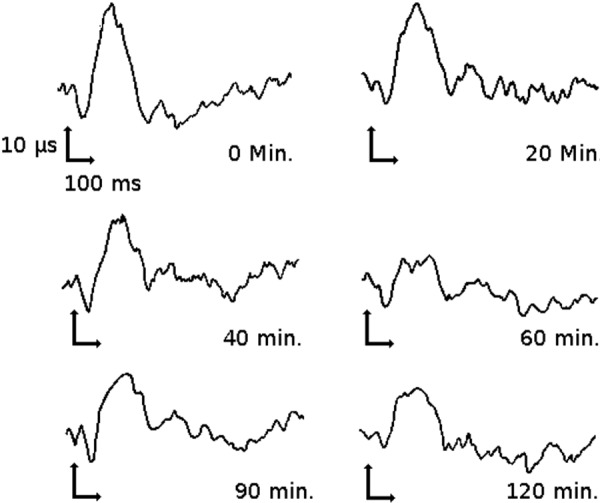
Change of SSEP in a subject after dosing with 500 mg acetylsalicylic acid (figure adapted from [94]).

**Figure 4.**
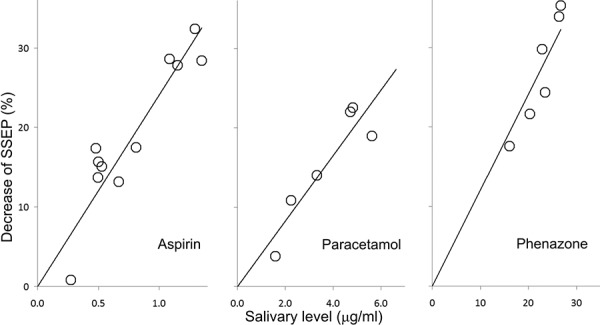
Correlation between percent SSEP reduction and acetylsalicylic acid concentration in the saliva (data obtained from [95]).

An example of such a study is described by Rohdewald et al. [95] where evoked potential amplitude induced by electrical stimulation of tooth pulp was used as a measure of analgesic effect. This study assessed the analgesic effect of acetylsalicylic acid in terms of SSEP and linked the effect to the saliva drug concentration profile. The resulting model produced a stronger linear correlation between evoked potential and saliva drug concentration than the correlation for the subjective measures, patient pain rating and minimum threshold of stimulation perception [95]. This alteration of SSEP by acetylsalicylic acid can be seen in Figure 3, and the relationship between SSEP decrease and saliva concentration of acetylsalicylic acid can be seen in Figure 4. 

An additional study utilized a similar approach to assess the analgesic effects of codeine using SSEPs and discovered a link with saliva codeine concentration [72]. 

### 4.2 Drugs used in anesthesia

General anesthetic drugs used to produce unconsciousness permit surgical procedures to be performed without a patient’s perception of pain or awareness [3]. Since chemical structures can vary significantly among anesthetics, a common method for classifying these drugs is route of administration. Namely, anesthetics can be broadly classified as i.v. anesthetics and inhaled anesthetics. 

**4.2.1 Intravenous anesthetics**

The most common i.v. anesthetics include thiopental, propofol, etomidate and ketamine [3]. The mechanism of action of thiopental, propofol and etomidate is GABAergic inhibition whereas ketamine exerts its effect via glutamatergic excitation [3]. 

Propofol has become increasingly popular over thiopental as the i.v. anesthetic of choice [3]. Propofol is a commonly used drug with many useful properties for intravenous anesthesia. The drug produces a rapid onset and offset of the hypnotic effect as it is quickly eliminated from the body [96]. Propofol’s anesthetic effects have been correlated with EEG using numerous techniques, including spectral-based EEG measures [97], EEG entropy estimations [40], BIS [98] and evoked potentials [99]. 

**4.2.2 Inhaled anesthetics**

**Figure 5:**
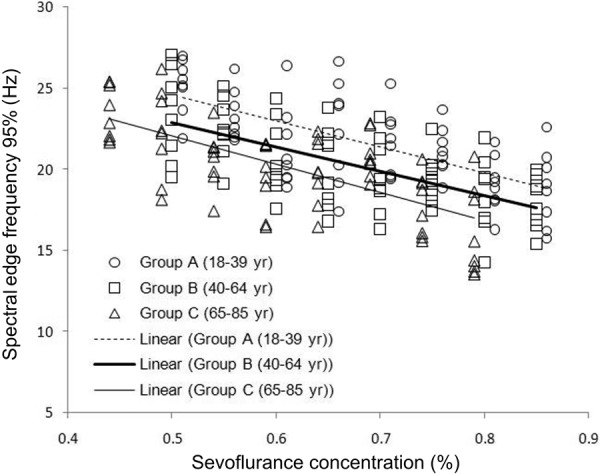
Spectral edge frequency (95%) versus end-tidal sevoflurane concentration in different age groups (data obtained and figure adapted from [102]).

Inhalation anesthetics are administered to the lungs in a gaseous form. Rather than a dosage, inhalation anesthetics are applied by setting a specific gas concentration [3]. When steady-state is reached, the gas concentration in the lung correlates with the brain concentration [3]. The actions of various inhaled anesthetics have been linked with EEG measures. Examples include correlation of desflurane levels with BIS [100], evoked potentials [100] and EEG Shannon entropy using effect-site concentrations derived from end-tidal concentrations [31]. In addition, sevoflurane effect-site concentrations (derived from end-tidal concentrations) have been pharmacodynamically linked with multiple EEG entropy measures including permutation entropy [29, 30], spectral entropy [32, 33], “state entropy” (calculated over 0.8 – 23 Hz frequency range) and “response entropy” (calculated over the frequency range of 0.8 – 47 Hz) [34]. Rehberg et al. [101] showed a strong correlation between the 95% spectral edge frequency and the effect site concentration derived from end-tidal concentrations of desflurane, isoflurane and sevoflurane. Furthermore, Katoh et al. [102] demonstrated that the 95% spectral edge frequency measure is not sensitive to patient age and may predict sedation depth better than end-tidal sevoflurane concentration. An example of the relationship of the 95% spectral frequency to the end-tidal sevoflurane concentration can be seen in Figure 5. 

### 4.3 Opioids

Opioids are a class of drugs commonly used for pain relief or to prepare patients for anesthesia [3]. Opioids produce their therapeutic effect by interacting with the µ-, d-, and k-receptors [103]. Opioids can serve as full agonists (such as morphine and methadone), partial agonists (such as pentazocine and butorphanol) or antagonists (such as naloxone and naltrexone) [3]. While opioids are clinically useful for the treatment of moderate to severe pain, the euphoric sensation produced is likely one reason for the high potential of abuse [3]. 

Opioids have been shown to produce a signature effect on EEG recordings. In particular, many opioids such as morphine, alfentanil, fentanyl, sufentanil, butorphanol, nalbuphine have been shown to alter the spectral power in the δ-range (0.5 – 4.5 Hz) [104]. In addition, the spectral edge frequency (95%) has been a useful surrogate measure of opioid drug effect [105]. Furthermore, opioids have been shown to produce an effect on evoked potentials. In particular, studies have shown that opioids affect the mid-latency auditory evoked response [106]. Remifentanil (also known as GI 87084B) has a well-established concentration-EEG relationship [105, 107, 108]. 

### 4.4 Anticonvulsants

Anticonvulsants, a class of drugs commonly used to treat seizures, can exert their effect in numerous ways. Some anticonvulsants make the neurons less excitable while others make the neural discharges less likely to propagate. The main types include sodium channel blockers, calcium current inhibitors, gamma-aminobutryic acid (GABA) enhancers, glutamate blockers, carbonic anhydrase inhibitors, hormones, as well as drugs with unknown mechanisms of action [109]. Smith et al. [76] demonstrate that lamotrigine and topiramate both affect EEG as well as auditory evoked potentials. An extensive review of anticonvulsant drugs is outside of this article’s scope. However, benzodiazepines can be used to treat status epilepticus and are an important drug class in the field of EEG pharmacokinetic/pharmacodynamic modeling. 

### 4.5 Benzodiazepines

Benzodiazepines (BZDs) are a class of short-acting drugs that target the inhibitory neurotransmitter receptors activated by GABA [110]. A BZD drug binds to the GABA_A_ receptor, which mediates chloride channel activity [110]. The chloride ion current modulation thereby enhances inhibitory neurotransmission to produce the therapeutic effect [111]. BZDs are often used in anesthetic applications as well as epilepsy treatment. Due to the sedative effects, BZDs may be more useful for acute (e.g. status epilepticus) rather than chronic seizure treatment. 

**Figure 6:**
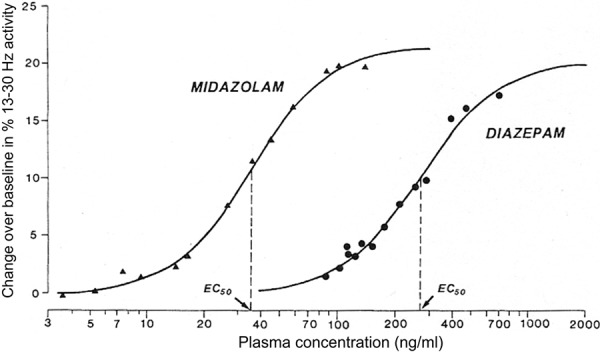
Relationship between diazepam or midazolam plasma concentration and the 13 – 30 Hz (β) spectral power change from baseline following intravenous doses (reprinted with permission, [114]). Each point represents the mean value for 11 study subjects. The solid lines represent the predicted concentrations based on a sigmoid E model fit to the data.

BZDs produce an EEG spectral power change in the β-band (13 – 30 Hz) corresponding with plasma concentrations and have been well-studied in both human and animal models [112]. Midazolam [113, 114, 115, 116], diazepam [114], flunitrazepam [113], oxazepam [113] and clobazepam [113] are examples of BZDs that show the aforementioned plasma concentration-EEG effect. Figure 6 is a prime example of the precise relationship between β-band EEG power and the plasma concentration of midazolam and diazepam [114]. 

**Figure 7:**
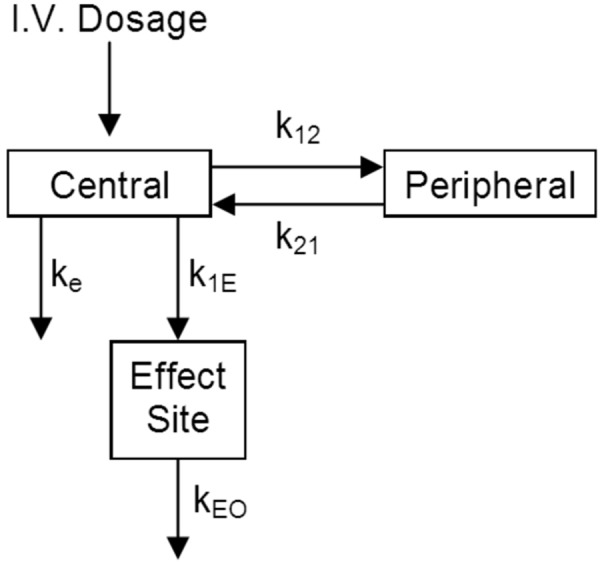
A two-compartment pharmacokinetic model coupled with an effect site used to model the EEG effect of lorazepam in healthy human volunteers after intravascular bolus and infusion administrations (figure adapted from [117]).

**Figure 8:**
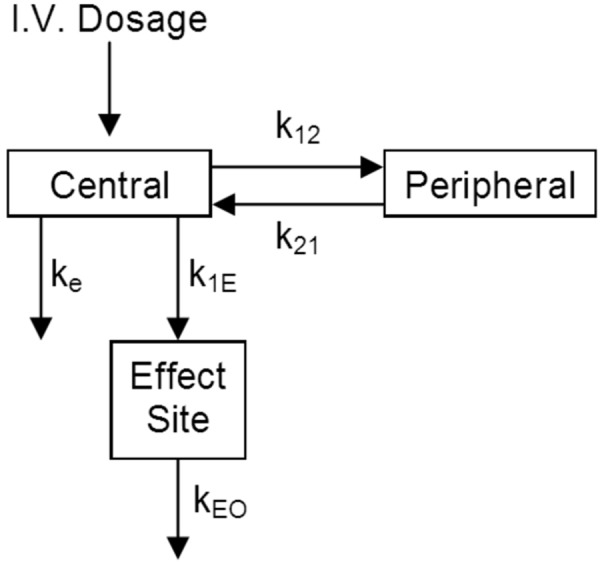
A: Changes over baseline in percent 13 – 30 Hz spectral power in relation to plasma lorazepam concentration in a human volunteer. The direction of increasing time goes along the path from the top right to the bottom left and demonstrates a counter-clockwise hysteresis. B: Changes over baseline in percent 13 – 30 Hz spectral power in relation to hypothetical effect-site lorazepam concentration in the same subject, where the solid line represents the sigmoid E model of the data (data obtained and figure adapted from [117]).

Greenblatt et al. [117] were able to model a relationship between the 13 – 30 Hz EEG spectral power and lorazepam plasma concentrations in healthy human volunteers following intravascular bolus and infusion administration. Using a 2-compartment model coupled to an effect site (Figure 7), the effect site concentration produced a useful correlation with the 13 – 30 Hz EEG spectral power (in contrast to the apparent lag or hysteresis when using plasma concentration, as shown in Figure 8). 

Some of the major challenges involved with prediction from such models are the dependence on route of administration as well as formulation of active metabolites [112]. However, the formulations for such models may serve as a useful tool for BZD drug development. 

In addition to individual drug effects, other studies have been able to express the interaction among multiple drugs using EEG. An example of such a study is the work by Jonker et al. [118] to assess the pharmacodynamic interaction between midazolam (an allosteric modulator for the GABA_A_-type receptor) and the anticonvulsant tiagabine (which inhibits the uptake of GABA at the synapse). The experiments demonstrated that midazolam and tiagabine express an additive in-vivo pharmacodynamic interaction, rather than synergistic. 

### 4.6 Antidepressants

Antidepressant efficacy is difficult to accurately quantify with consistency. Subjective measuring methods such as polling patients for their perceived improvement after treatment may result in a large degree of interindividual and intraindividual variability. On the other hand, more biologically relevant measures such as neurotransmitter concentrations can be extremely difficult and expensive to measure from various brain regions over time. Though there are some noted inconsistencies in EEG effects among various antidepressant drugs [119], the objectivity and non-invasive nature of scalp-EEG make it a desirable tool for quantifying the pharmacological action. 

One such method that has demonstrated success at objectively quantifying the physiologic effect of antidepressants is cordance (see Section 3.1.3 for details). EEG Cordance has been applied to the study of depression for venlafaxine [15, 17] and fluoxetine [15] in a dose-response manner. Cook et al. [120] demonstrated that subjects with the greatest cordance changes after fluoxetine or venlafaxine treatment produced the most complete 8-week responses in adult patients with unipolar depression. These studies demonstrate potential for EEG-based prediction of antidepressant drug response. 

The P300 evoked potential has also demonstrated sensitivity to antidepression pharmacotherapy. Anderer et al. [51] demonstrated that the antidepressant citalopram produced an increase in the P300 source strength (evoked with auditory stimuli) in the left prefrontal cortex and extending into the posterior cingulate compared to placebo using the LORETA current source density estimation method. The study determined that a P300 source strength reduction was detected bilaterally in temporal and medial prefrontal regions (extended to rostral portions of the anterior cingulate) in menopausal depressed patients compared to age-matched controls. 

In addition, numerous studies demonstrate antidepressant effects on the EEG spectrum. Arnold et al. [121] demonstrated that S-adenosyl-l-methionine produced an increase in total EEG power, a decrease in relative and absolute power in the slow α- and δ/θ-frequencies, an increase in relative and absolute power in the β- and α-2 frequencies, and an acceleration of the centroids of the α and total power spectrum in elderly normal volunteers. Bruder et al. [122] demonstrated that depressed patients whom were considered responders (“much or very much improved” as measured by the Clinical Global Impression Improvement scale) to fluoxetine produced a greater α-response over the right occipital region compared to the left region. The non-responders tended to show an opposing asymmetry. Leuchter et al. [123] applied an EEG index measure called the Antidepressant Treatment Response index (ATR, Aspect Medical Systems; Norwood, MA) which was able to predict the response as measured by the 17-item Hamilton Depression Rating Scale for escitalopram and bupropion, but not the combined therapy in subjects with major depressive disorder. The ATR index is a weighted sum of relative α- and θ-power at Week 1 and the difference between baseline α-1 power and α-2 power at Week 2, transformed to range from 0 to 100 (low probability to high probability of response, respectively) [124].[Table Table1]

### 4.7 Antipsychotics

Antipsychotics are approved for treating schizophrenia, mood episodes such as depression or acute mania, and have been used “off-label” for psychotic symptoms such as those occurring in delusional disorder, delirium, Parkinson’s disease, psychotic depression and dementia [125]. This class of drugs is coarsely sub-divided into typical and atypical antipsychotics. Some examples of typical (“first generation”) antipyschotics include chlorpromazine, haloperidol and zuclopenthixol while atypical (“second generation) antipsychotics include amisulpride, clozapine, olanzapine, quetiapine, risperidone and ziprasidone [126]. Drug-induced EEG effects arising from drugs in both antipsychotic drug classes have been reported in the literature. 

Lee et al. [127] applied pharmacokinetic/pharmacodynamic modeling to link risperidone effect compartment concentrations to EEG measures in healthy young males. The absolute power in the δ- and θ-bands after risperidone treatment was greater than placebo for all EEG channels. A study by Kim et al. [128] analyzed the effect of polymorphisms of the cytochrome P450 2D6 (CYP2D6) and dopamine D2 receptor genes on EEG power response to aripiprazole in healthy male volunteers. While the DRD2 genotypes studied did not influence the EEG, the *1/*5 and *1/*10 CYP2D6 alleles demonstrated a trend towards increased area under the aripiprazole concentration time curve which had a linear relationship to the area under the EEG response versus time curve for absolute δ-power in the Cz channel. 

A recent study by Romero et al. [129] examined the effects of ocular filtering on the EEG effects of risperidone, olanzapine, and haloperidol in healthy volunteers. The effects included EEG spectral power increases (in α- and combined δ/θ-powers after risperidone administration as well as relative and absolute δ-powers after olanzapine administration) and decreases (in the combined δ-θ centroid after haloperidol administration as well as combined δ/θ-centroid and absolute α-power after olanzapine administration) that were both augmented by application of the BSS-based ocular artifact removal method. 

Tislerova et al. [130] examined the effects of clozapine, olanzapine, and risperidone in patients with schizophrenia using LORETA functional imaging. Overall, antipsychotic-naive schizophrenic subjects receiving any of the study drugs generally demonstrated an increase in slow δ- and θ-frequencies over the fronto/temporo/occipital cortical region, an increase in α-1 and α-2 at the temporal cortex, and an increase in β-1 and β-2 at the temporo/occipital and posterior limbic region compared to healthy subjects receiving the same treatment. Patients receiving clozapine demonstrated an increase in θ- and δ-frequencies in the medial frontal cortex and anterior cingulate and a decrease in occipital α-1 and β-2 compared to antipsychotic naïve patients. Patients receiving olanzapine produced an increase in θ-frequencies at the anteriror cingulum as well as a decrease in occipital cortical and posterior limbic α-1, β-2, and β-3 and a decrease in β-3 at the anterior cingulum and frontotemporal cortex compared to antipsychotic naïve patients. Finally, patients receiving risperidone did not demonstrate any identifiable EEG changes from the antipsychotic naïve group. 

It is important to note that inconsistencies in EEG effects among antipsychotic drugs have been reported [119]. Furthermore, in spite of the numerous studies citing risperidone-induced EEG effects, Allain et al. [131] did not observe any risperidone-induced EEG alterations in healthy elderly volunteers. In particular, risperidone 25 or 50 mg did not produce statistically significant differences in δ-, θ-, α-, β-, as well as total EEG power when compared to placebo. While some interesting treatment-related EEG effects have been reported, the inconsistent and conflicting study results suggest that additional research may be required before EEG-based effect measures can be applied to antipsychotic drug development. 

## 5. Future directions / potential applications

The wide availability, non-invasive operation, and potential cost savings make EEG a desirable choice for a surrogate effect measure of drugs that act on the central nervous system. This paper provides a survey of some common pharmacodynamic modeling approaches using EEG measures. 

Some additional EEG-based pharmacodynamic research directions may include assessment of a compound’s pharmacological characteristics, comparison of potencies and efficacies among drugs, tolerance development, the role of metabolites, enantiomers, drug-drug interactions, circadian rhythms, intrinsic and extrinsic factors affecting an observed effect, and incorporation of physiopathological drug interaction mechanisms [132]. 

One area for improvement is the characterization of drug-induced EEG changes in pediatric patients. Jeleazcov et al. [133] describes how EEG variables performed less successfully as arousal measures in infants (age 0 – 1 year) than children (age 1 – 13 years) undergoing surgical anesthesia with remifentanil and propofol. Davidson et al. [134] determined that in contrast to older children, no changes in power or 90% spectral edge frequency were observed in infants emerging from anesthesia for various combinations of anesthetics. Furthermore, the authors stated a need to enhance EEG-based arousal measure algorithms for infants should EEG monitoring of anesthesia depth be utilized in this patient population. 

From a technological perspective, the state of the art for computer hardware provides cost-effective data processing and storage solutions making EEG research more affordable and manageable than ever before. As time goes on, EEG acquisition hardware improvements such as increased sampling rate, sampling precision, number of channels, and preprocessing options may help improve the accuracy of EEG-based pharmacodynamic models. 

Overall, neuroactive drug development could benefit from pharmacokinetic/pharmacodynamic modeling using EEG features as surrogate drug effects. In order for this approach to succeed, such a model should be backed by substantial supportive evidence. For example, both the BZD drug class [112, 113, 114, 115, 116, 117, 118] and opioid drug class [104, 105, 106, 107, 108] have demonstrated a consistent EEG-concentration relationship that is well-characterized by pharmacodynamic models. The benefits of such a robust EEG-based pharmacodynamic model for drug development can include expediting clinical trials, enhanced clinical trial safety, reduction in clinical trial cost, or a combination of the three. For example, the EEG-concentration relationship established for BDZs and opioids could help rapidly determine doses for clinical trials. While the other EEG-based pharmacodynamic studies cited in this paper appear promising, subsequent research must be performed to validate the proposed EEG-based measures and/or the corresponding pharmacodynamic models before such findings may be considered appropriate for drug development. 

## Acknowledgments

This work was supported in part by NIH grant 1UL1RR029890 (Clinical and Translational Science Award), the University of Florida Clinical Translational Science Institute (CTSI), and the University of Florida Department of Pharmaceutics. 

**Equation 1.**
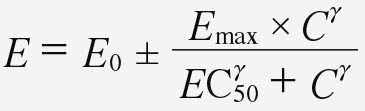
Equation 1.

**Equation 2. Equation2:**
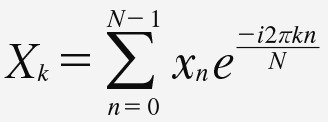
Equation 2.

**Equation 3. Equation3:**
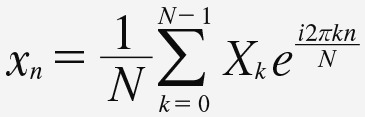
Equation 3.

**Equation 4. Equation4:**
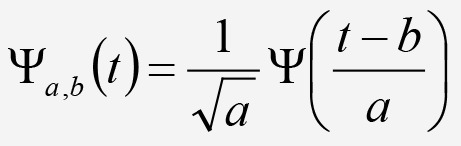
Equation 4.

**Equation 5. Equation5:**
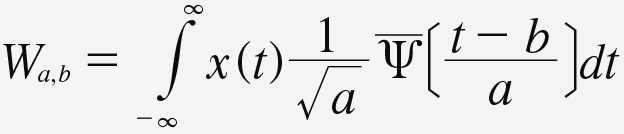
Equation 5.

**Equation 6. Equation6:**
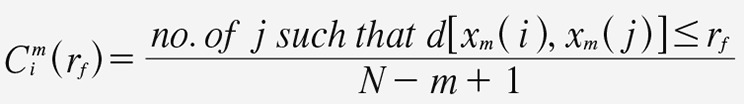
Equation 6.

**Equation 7. Equation7:**
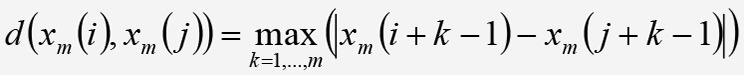
Equation 7.

**Equation 8. Equation8:**
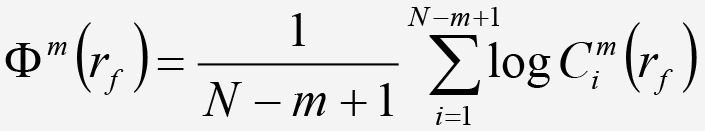
Equation 8.

**Equation 9. Equation9:**
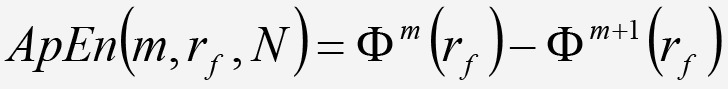
Equation 9.

**Table 1 Table1:** Summary of studies that utilize EEG-based pharmacodynamic measures. δ = the delta band of the EEG, θ = the theta band of the EEG, α = the alpha band of the EEG, β = the beta band of the EEG, SSEP = somatosensory evoked potential, AEP = auditory evoked potential, LORETA = low-resolution brain electromagnetic tomography, SEF n% = Spectral edge frequency for n% of the spectral power, BIS = bispectral index.

Class	Drug	δ	θ	α	β	SSEP	AEP	LORETA current source density	SEF n%	BIS	Other	Citation
Analgesic	Acetylsalicylic acid					x						[95]
	Alfentanil	x										[104]
	Butorphanol	x										[104]
	Codeine					x						[72]
	Fentanyl									x		[47]
	Fentanyl	x										[104]
	Morphine	x										[104]
	Nalbuphine	x										[104]
	Remifentanil									x	Approximate entropy	[40]
	Remifentanil								95	x	Approximate entropy	[97]
	Remifentanil										Nonlinear regression	[107]
	Remifentanil								95			[108]
	Remifentanil	x	x	x	x				25, 50, 75, 95	x	Approximate entropy, 26-32 Hz power, 32-47 Hz power	[133]
	Remifentanil								95			[105]
	Sufentanil	x										[104]
	Sufentanil						x					[106]
Anesthetic	Desflurane										Shannon entropy	[31]
	Desflurane								95			[101]
	Desflurane						x			x		[100]
	Isoflurane								95			[101]
	Propofol									x	Approximate entropy	[40]
	Propofol								95	x	Approximate entropy	[97]
	Propofol						x				Burst suppression	[99]
	Propofol	x	x	x	x				25, 50, 75, 95	x	Approximate entropy, 26 – 32 Hz power, 32 – 47 Hz power	[133]
	Propofol									x		[43]
	Propofol									x		[98]
	Propofol									x	Spectral entropy, state entropy, response entropy	[35]
	Sevoflurane										State entropy response entropy	[34]
	Sevoflurane								95			[102]
	Sevoflurane										Permutation entropy	[29]
	Sevoflurane										Spectral entropy	[32]
	Sevoflurane										Spectral entropy	[33]
	Sevoflurane										Permutation entropy	[30]
	Sevoflurane								95			[101]
Antidepressant	Ademetionine						x	x				[51]
	Ademetionine	x	x	x	x							[121]
	Bupropion		x	x								[123]
	Citalopram						x	x				[51]
	Escitalopram		x	x								[123]
	Fluoxetine			x								[122]
	Fluoxetine	x	x	x	x						cordance	[120]
	Fluoxetine	x	x	x	x						cordance	[15]
	Venlafaxine	x	x	x	x						cordance	[17]
	Venlafaxine	x	x	x	x						cordance	[120]
	Venlafaxine	x	x	x	x						cordance	[15]
Antipsychotic	Aripiprazole	x										[128]
	Clozapine	x	x	x	x			x				[130]
	Haloperidol	x	x									[129]
	Olanzapine	x	x	x								[129]
	Olanzapine		x	x	x			x				[130]
	Risperidone	x	x									[127]
	Risperidone	x	x	x								[129]
Anxiolytic	ABIO-08/01	x	x	x	x							[55]
	ABIO-08/01							x				[56]
	Buspirone	x	x	x	x			x				[50]
	Clobazepam				x							[113]
	Diazepam				x							[114]
	Diazepam										Interictal spikes	[59]
	Flunitrazepam				x							[113]
	Lorezpam				x							[117]
	Midazolam									x		[47]
	Midazolam				x							[114]
	Midazolam				x							[116]
	Midazolam										Interictal spikes	[59]
	Midazolam				x							[115]
	Midazolam				x							[113]
	Oxazepam				x							[113]
	Suriclone	x	x	x	x							[54]
